# Human antibody reaction against recombinant salivary proteins of *Phlebotomus orientalis* in Eastern Africa

**DOI:** 10.1371/journal.pntd.0006981

**Published:** 2018-12-04

**Authors:** Petra Sumova, Michal Sima, Tatiana Spitzova, Maha E. Osman, Anderson B. Guimaraes-Costa, Fabiano Oliveira, Dia-Eldin A. Elnaiem, Asrat Hailu, Alon Warburg, Jesus G. Valenzuela, Petr Volf

**Affiliations:** 1 Department of Parasitology, Faculty of Science, Charles University, Prague, Czech Republic; 2 Commission for Biotechnology and Genetic Engineering, National Centre for Research, Khartoum, Sudan; 3 Vector Molecular Biology Section, Laboratory of Malaria and Vector Research, National Institute of Allergy and Infectious Diseases, National Institutes of Health, Rockville, Maryland, United States of America; 4 Department of Natural Sciences, University of Maryland Eastern Shore, Princess Anne, Maryland, United States of America; 5 Department of Microbiology, Immunology and Parasitology, College of Health Sciences, Addis Ababa University, Addis Ababa, Ethiopia; 6 Department of Microbiology and Molecular Genetics, The Kuvin Centre for the Study of Infectious and Tropical Diseases, The Hebrew University—Hadassah Medical School, The Hebrew University of Jerusalem, Jerusalem, Israel; Instituto de Ciências Biológicas, Universidade Federal de Minas Gerais, BRAZIL

## Abstract

**Background:**

*Phlebotomus orientalis* is a vector of *Leishmania donovani*, the causative agent of life threatening visceral leishmaniasis spread in Eastern Africa. During blood-feeding, sand fly females salivate into the skin of the host. Sand fly saliva contains a large variety of proteins, some of which elicit specific antibody responses in the bitten hosts. To evaluate the exposure to sand fly bites in human populations from disease endemic areas, we tested the antibody reactions of volunteers' sera against recombinant *P*. *orientalis* salivary antigens.

**Methodology/Principal findings:**

Recombinant proteins derived from sequence data on *P*. *orientalis* secreted salivary proteins, were produced using either bacterial (five proteins) or mammalian (four proteins) expression systems and tested as antigens applicable for detection of anti-*P*. *orientalis* IgG in human sera. Using these recombinant proteins, human sera from Sudan and Ethiopia, countries endemic for visceral leishmaniasis, were screened by ELISA and immunoblotting to identify the potential markers of exposure to *P*. *orientalis* bites. Two recombinant proteins; mAG5 and mYEL1, were identified as the most promising antigens showing high correlation coefficients as well as good specificity in comparison to the whole sand fly salivary gland homogenate. Combination of both proteins led to a further increase of correlation coefficients as well as both positive and negative predictive values of *P*. *orientalis* exposure.

**Conclusions/Significance:**

This is the first report of screening human sera for anti-*P*. *orientalis* antibodies using recombinant salivary proteins. The recombinant salivary proteins mYEL1 and mAG5 proved to be valid antigens for screening human sera from both Sudan and Ethiopia for exposure to *P*. *orientalis* bites. The utilization of equal amounts of these two proteins significantly increased the capability to detect anti-*P*. *orientalis* antibody responses.

## Author summary

Hosts repeatedly bitten by phlebotomine sand flies develop species-specific antibody responses against certain sand fly salivary antigens. Salivary gland homogenate (SGH) is frequently used to evaluate the levels of this antibody response in host. However, SGH is less suitable for large-scale studies, since obtaining sufficient numbers of salivary glands is labor intensive and requires expertise in dissection. To replace SGH as antigen to screen for exposure to sand fly bites, specific recombinant salivary antigens were utilized. Our study assessed the human antibody reactions against recombinant salivary proteins of *Phlebotomus orientalis*. This sand fly species is a vector of *Leishmania donovani*, the causative agent of severe visceral leishmaniasis in Eastern Africa. To identify valid markers of exposure to *P*. *orientalis* in humans, we screened for anti-*P*. *orientalis* antibody responses in serum samples from individuals residing in Sudan and Ethiopia. We tested nine recombinant salivary antigens and found a combination of yellow-related protein (mYEL1) and antigen 5-related protein (mAG5) the best marker of exposure, accurately correlating with the levels of exposure to *P*. *orientalis* bites as determined using SGH. Thus the combination mYEL1+ mAG5 can comprise a useful epidemiological tool to determine levels of exposure to *P*. *orientalis* in populations living in endemic areas of Eastern Africa, which could help in monitoring the distribution of *P*. *orientalis* and therefore assessing suitable anti-vector campaigns.

## Introduction

Phlebotomine sand flies (Diptera: Phlebotominae) are blood sucking insects that transmit parasites of the genus *Leishmania* (Kinetoplastida: Trypanosomatidae). *Phlebotomus orientalis* is an important vector of *Leishmania donovani* in Sudan, Ethiopia and Kenya [1]. In these countries, *Le*. *donovani* is a causative agent of life-threatening visceral leishmaniasis (VL), with an estimated incidence reaching 39 thousand people annually [2]. The optimal strategy for controlling VL depends on understanding the epidemiology and transmission of *Le*. *donovani* by its vectors. Visceral leishmaniasis in Eastern Africa was believed to be anthroponotic, but recent findings of *Leishmania* DNA and seroprevalence against *Leishmania* antigen in domestic animals indicates the possibility of anthropozoonotic transmission [3–5]. Moreover, *P*. *orientalis* feeds on humans as well as on domestic and wild animals [3,6–8].

During blood-feeding, female sand flies salivates into the host skin. Saliva contains a cocktail of pharmacologically active proteins that facilitate successful blood feeding on host [9]. Some salivary proteins elicit antibody response, which may be species-specific [10,11] and can, therefore, serve as a marker of exposure to sand flies in bitten hosts [12]. In humans, the predominant subtypes of antibody responses to sand fly saliva differ between vector species [9]. For this reason, in most studies that determined the level of antibody responses to sand fly bites, authors measured basic IgG levels in sera of bitten people. The exposure of human populations to sand fly bites was evaluated through measuring specific antibody responses against salivary gland homogenate (SGH) of several sand fly species including *L*. *longipalpis* in Brazil [13,14], *P*. *sergenti* and *P*. *papatasi* in Turkey [15] and Israel [16], *P*. *papatasi* in Tunisia [17,18], *P*. *arabicus* in Israel [16], and *P*. *orientalis* in Ethiopia [8]. The aforementioned human antibody response against salivary proteins was also utilized to validate efficacy of vector control programs aimed against *P*. *argentipes* and *P*. *papatasi* in India [19,20].

These studies required laborious and time consuming dissections of large quantities of salivary glands. To make large-scale studies more feasible, the most antigenic salivary proteins need to be identified and expressed in recombinant form. Recombinant proteins have already proven their utility in large-scale studies screening anti-phlebotomine antibodies in human populations. In Brazil, recombinant salivary proteins LJM17 and LJM11 [21], and rLinB-13 [22] are able to detect anti-*L*. *longipalpis* and anti-*L*. *intermedia* antibodies, respectively. Similarly, a recombinant protein (rPpSP32) was used to screen for exposure to *P*. *papatasi* bites in Saudi Arabia and Tunis [23,24]. All four proteins were produced in a mammalian expression system and were demonstrated to be valid substitution for SGH in serological tests [21–23].

The current study is the first one aimed at validation of antigenic properties of *P*. *orientalis* salivary proteins produced in different expression systems, and evaluating their potential to serve as a substitution for SGH in serological surveys screening exposure to *P*. *orientalis* bites in humans from endemic areas of Eastern Africa.

## Methods

### Ethical statement

Written informed consent was obtained from all the adult volunteers who participated in the study and for inclusion of young children written consent was obtained from their parents or guardians. For Ethiopian samples, the study protocols were reviewed and approved by the ethical review committee at the Medical Faculty, Addis Ababa University and the National Research Ethics Review Committee (NRERC) at the Ethiopian Ministry of Science and Technology. For Sudanese samples, the study protocols were approved by the National Ethical Committee of the federal Ministry of Health, Sudan. Each volunteer was treated in accordance with international bio-ethical rules and laws for human sampling. Blood was sampled by certified trained Health officers or nurses.

### Serum samples

Fifty human serum samples from Sudan and 235 samples from Ethiopia were used for specific anti-*P*. *orientalis* antibody detection. Eighteen serum samples were used as negative controls: i) nine samples were collected in USA from volunteers with known travel history. and ii) nine human serum samples were collected from residents of Khartoum (Sudan), where *Phlebotomus orientalis* is not present. All sera were obtained from volunteers which did not travel to locations were *P*. *orientalis* is abundant. Sera were stored at -80°C and aliquoted before use.

### Sand flies and salivary gland homogenate

A laboratory colony of *P*. *orientalis* originating from Ethiopia (established in 2008) [25] was reared under standard conditions [26]. Salivary glands of *P*. *orientalis* were dissected from 4–6 day old female sand flies into 20mM Tris buffer with 150mM NaCl (20 glands per 20 μl of buffer) and stored at -80°C. Before use, salivary glands were disrupted by three freeze-thawing cycles in liquid nitrogen into salivary gland homogenate (SGH).

### Recombinant proteins

Nine recombinant proteins based on six different *P*. *orientalis* salivary antigens were prepared in bacterial (two proteins), mammalian (one protein), or both (three proteins) expression systems (Table 1) and used as antigens for detection of anti-*P*. *orientalis* IgG in human sera. Bacterially expressed proteins were selected based on their antigenicity in ELISA using domestic animal sera from Ethiopia [11]. The most antigenic proteins recognized by antisera from domestic animals (YEL1 and PAR25) were expressed also in mammalian system. The second yellow-related protein (mYEL2) was expressed in mammalian system due to its sequence similarity to mYEL1 (78%). Mammalian AG5 protein was prepared based on the reactivity of the native protein from SGH on immunoblot with human sera from Sudan and Ethiopia.

**Table 1:** Recombinant *P*. *orientalis* salivary proteins.

Name	Protein family	Expression system	GenBank ACCN
**bAPY**	Apyrase	*E*. *coli*	AGT96431
**bYEL1**	Yellow-related	*E*. *coli*	AGT96428
**bPAR25**	ParSP25-like	*E*. *coli*	AGT96466
**bD7**	D7-related	*E*. *coli*	AGT96467
**bAG5**	Antigen 5-related	*E*. *coli*	AGT96441
**mYEL1**	Yellow-related	HEK293	AGT96428
**mYEL2**	Yellow-related	HEK293	AGT96427
**mPAR25**	ParSP25-like	HEK293	AGT96466
**mAG5**	Antigen 5-related	HEK293	AGT96441

List of nine recombinant proteins based on salivary antigens of *P*. *orientalis*. Designation, protein families, expression systems and GenBank accession numbers are indicated.

The bacterial forms of the recombinants with His-tag were expressed in *Escherichia coli* BL21 (DE3) gold cells (Agilent) and purified using Ni-NTA column (Bio-Rad) as described in [11]. Mammalian counterparts of these proteins were produced and purified as described elsewhere [27]. Briefly, the synthetic DNA fragments (GeneArt Strings, ThermoFisher Scientific) coding recombinant proteins including His-tag at the C terminal end were cloned into VR2001-TOPO vector [28] and transformed by heat shock in TOP-10 cells (ThermoFisher Scientific). Plasmids were isolated and sent to Leidos, NCI, Protein Expression Laboratory (Frederick, MD) for transfection and expression. Transfected FreeStyle human embryonic kidney 293-F cell cultures (HEK293) were harvested after 72 h. Supernatant was concentrated from 2 liter to 400 ml using a Stirred Ultrafiltration Cell unit (Millipore) with ultrafiltration membrane (Millipore). Recombinant proteins were purified by a HPLC system (Bio-Rad) using a HiTrap Chelating HP columns (GE Healthcare Biosciences) charged with nickel by a gradient of imidazole. Protein concentration was measured using a NanoDrop ND-1000 (ThermoFisher Scientific) spectrophotometer at 280 nm and calculated using the extinction coefficient of the protein. Identity and high purity of the proteins was verified by mass spectrometry.

### Study design

To determine the best candidates among recombinant proteins, experiments were conducted in several subsequent steps. In the first one, human serum samples from individuals living in areas where *P*. *orientalis* was prevalent were tested by ELISA with *P*. *orientalis* SGH as antigen to detect the levels of specific IgG antibodies in these sera. The most antigenic *P*. *orientalis* salivary proteins were defined by immunoblot. To determine their antigenicity, all nine recombinant proteins were tested by ELISA using five different serum samples (three anti-*P*. *orientalis* SGH positive and two anti-*P*. *orientalis* SGH negative (2^nd^ step). The promising recombinants were used in ELISA with twenty human sera displaying varying anti-*P*. *orientalis* SGH reactivity and five non-exposed human sera controls (3^rd^ step). Several combinations of antigen-sera dilutions were tested to determine the optical density (OD) values matching in a best way with anti-SGH. In the final series of experiments (4^th^ step), only the most promising recombinant proteins were tested on immunoblot with five human sera, and by ELISA with all available samples. The dilutions of antigens and sera in each set of ELISA experiments are indicated in S1 Table.

### ELISA

ELISA plates (ThermoFisher Scientific) were coated with SGH (28 ng of proteins per well; corresponding to 0.2 gland per well) or recombinant proteins (exact amounts specified in S1 Table) diluted in 20mM carbonate-bicarbonate buffer (pH 9.5) at 4°C overnight. After washing with phosphate-buffered saline with 0.05% Tween 20 (PBS-Tw), plates were blocked with 6% non-fat dried milk (Bio-Rad) diluted in PBS-Tween and incubated for one hour at 37°C. After another washing step, plates were incubated with sera diluted in 2% non-fat dried milk for 1.5 hour at 37°C (sera dilutions specified in S1 Table). Plates were washed and incubated for 45 minutes at 37°C with peroxidase-conjugated Anti-Human IgG antibody (Sigma-Aldrich) diluted 1:1000 in PBS-Tween. The chromogenic reaction was developed for six minutes in McIlwain phosphate-citrate buffer (pH 5.5) with OPD (orthophenylendiamine, Sigma-Aldrich) and hydrogen peroxide in dark. The reaction was stopped by adding 10% sulfuric acid and measured at 492 nm using a Tecan Infinite M200 microplate reader (Schoeller). In each step, 100 μl of solution was used and each sample was tested in replicates.

### Immunoblot

The immunogenicity of the *P*. *orientalis* salivary proteins was tested by the immunoblot technique. Salivary gland homogenate (4.2 μg of total salivary proteins per lane, equivalent to 30 glands) and recombinant proteins mAG5, mYEL1, and mAG5 + mYEL1 (3 μg of each protein per lane) were electrophoretically separated on a 12% polyacrylamide gel under non-reducing conditions using a Mini-protean apparatus (Bio-Rad). Separated protein bands were transferred onto a nitrocellulose membrane using the iBLOT system (Invitrogen) and then cut into strips. Strips were either stained with amidoblack (Merck; 0.1% solution in 25% isopropanol and 10% acetic acid) or blocked in 5% non-fat milk diluted in Tris-buffered saline with 0.05% Tween 20 (TBS-Tw) overnight at 4°C and subsequently incubated for 1 hour with human sera diluted 1:50 in TBS-Tw. After the washing step with TBS-Tw, the strips were incubated with peroxidase-conjugated Anti-Human IgG Antibody (Sigma-Aldrich) diluted 1:500 in TBS-Tw. The chromogenic reaction was developed using a substrate solution containing diaminobenzidine and H_2_O_2_.

### Statistical analysis

The OD values of anti-SGH antibodies were used as the gold standard to validate the recombinant proteins in ELISA tests by calculating the respective positive and negative predictive values (PPV and NPV, respectively), sensitivity, and specificity. The cut-off value was calculated as the average OD of negative controls plus three times the standard deviation of negative controls. All serum samples were used in duplicates, averages of measured ODs were calculated and the blank value (average OD from wells without antigen, antibodies, and conjugate) was subtracted from each sample. Statistical analyses were carried out using R software (http://cran.r-project.org/). The non-parametric Spearman rank correlation test was used to assess correlations between total anti-SGH and anti-recombinant proteins IgG levels. To compare medians between locations and antigens, the non-parametric Kruskal-Wallis test and as a post-hoc test Wilcoxon rank sum test with Holm correction were used. A P-value of < 0.05 was considered to indicate statistical significance. The results were graphically presented using „ggplot2”package in R software [29].

## Results

### Human sera of individuals from endemic areas recognize *P*. *orientalis* SGH

Human sera (303 samples) were tested in duplicates with SGH of *P*. *orientalis* (S1 Fig). The ELISA cut-off value (0.238) was calculated from 18 negative controls. The positivity or negativity of all samples was defined based on the cut-off value. In total, 52% of the sera samples from Sudan and 34.5% samples from Ethiopia were determined as anti-*P*. *orientalis* positive. Median OD values for Sudan and Ethiopia were 0.25 (range: 0.06–1.6) and 0.19 (range: 0.04–1.4), respectively. When compared to the median value of negative controls (0.1, range: 0.04–0.19) they differed significantly (p < 0.0001). The difference in anti-*P*. *orientalis* antibody levels between Sudan and Ethiopia was marginally significant (p = 0.08).

### Immunoblot with SGH and human sera

**Fig 1:**
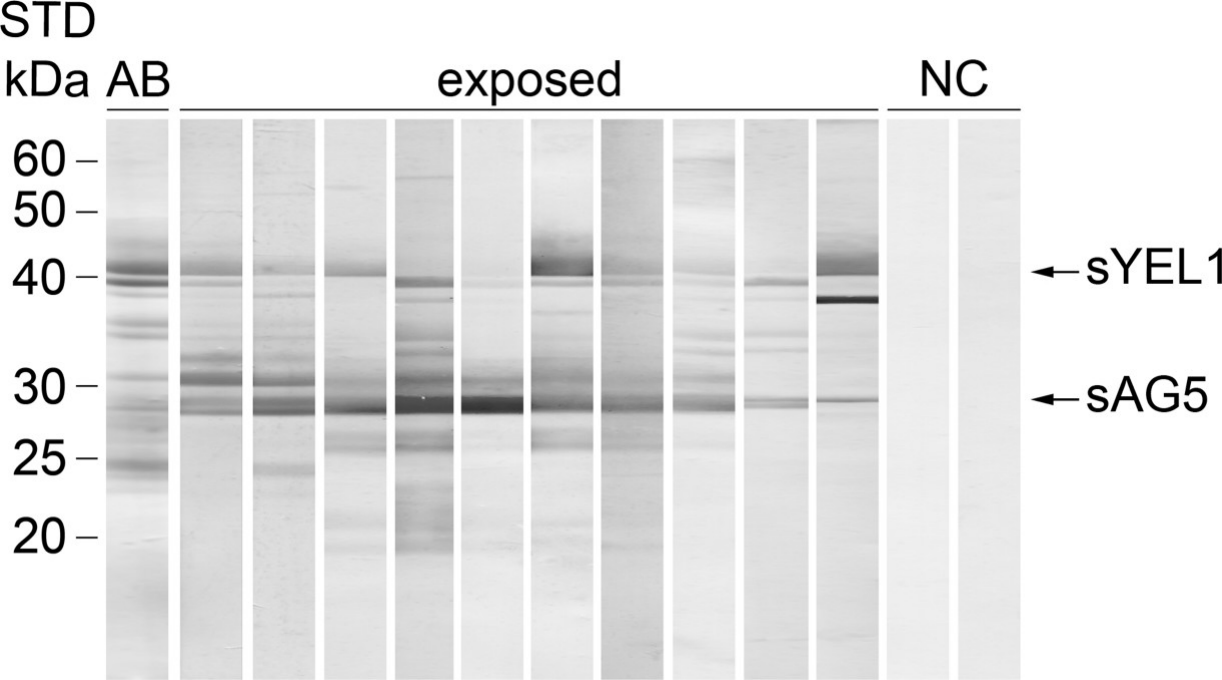
Immunoblot profile of *P*. *orientalis* salivary antigens. *P*. *orientalis* salivary proteins were electrophoretically separated under non-reducing conditions. Western blot analysis was performed with ten anti-SGH ELISA positive human sera (exposed) and two negative sera (NC). AB stands for salivary gland protein profile stained by amidoblack; sYEL1 and sAG5 stands for salivary yellow-related protein 1 and salivary antigen 5-related protein, respectively. Molecular weights (kDa) of standard (STD; BenchMark Protein Ladder, ThermoFisher Scientific) are indicated.

Antigenic *P*. *orientalis* salivary proteins were identified by immunoblot (Fig 1). In total, 10 highly reactive human serum samples from Sudan and Ethiopia and two negative samples from USA were chosen according to their optical densities in anti-SGH ELISA. Anti-*P*. *orientalis* positive serum samples recognized up to 10 protein bands with different intensities. The most prominent bands of approximately 40 kDa and 28 kDa corresponded to the molecular weight of the yellow-related protein (sYEL1) and antigen 5-related protein (sAG5), respectively. These two proteins were recognized by the majority of sera samples.

### Assessment of antigenic properties of recombinant proteins

In total, six different *P*. *orientalis* salivary proteins were expressed in recombinant form. Five proteins (bAPY, bYEL1, bPAR25, bD7, bAG5) were expressed in a bacterial system; three of their counterparts (mYEL1, mPAR25, mAG5) as well as a second *P*. *orientalis* yellow-related protein mYEL2, were expressed in a mammalian system. In the preliminary experiments using ELISA, antigenic properties of all proteins were tested with five human serum samples. Three different dilutions of antigens and two dilutions of sera (S1 Table: 2^nd^ step) were used to assess the optimal concentration for more extensive experiments. For recombinant proteins expressed in the mammalian system, lower concentrations were used owing to higher purity of these proteins. Results are summarized graphically in S2 Fig.

Based on these initial results, five proteins were excluded from further experiments, the reason was either no difference in OD of anti-SGH positive/negative sera (bAG5, bYEL1, bD7) or false positivity in negative sera (bAPY, mYEL2). For four promising proteins which were subsequently used for further experiments (bPAR25, mPAR25, mYEL1 and mAG5), the lowest eligible concentrations and sera dilutions were selected (S1 Table: 3^rd^ step).

### Evaluation of promising recombinant antigens using ELISA

Four promising antigens (bPAR25, mPAR25, mYEL1 and mAG5) were tested by ELISA with 20 Sudanese human serum samples covering the range of anti-SGH positivity/negativity and with five negative control samples from USA and Sudan. Apart from mYEL1, all proteins were tested in single concentration and sera dilution. For mYEL1, two different sera dilutions were tested to justify usage of lower dilution (1:100). Results are summarized in S2 Table. Recombinant ParSP25-related protein expressed in both bacterial and mammalian system was excluded from more extensive experiments due to high cut-off values, low correlation coefficient and big difference in serum positivity in comparison to SGH. Antibody response against mYEL1 correlated well with antibody reaction against SGH in both sera dilution, but the cut off and median values were slightly higher in dilution 1:50, which led to subsequent use of dilution 1:100. Together with mYEL1, antibody reaction against mAG5 also corresponded to anti-SGH response and both proteins showed low cut off values. These two antigens were therefore further analyzed as promising candidates for assessing exposure of human populations to *P*. *orientalis* bites.

### Immunoblot with recombinant salivary proteins mYEL1 and mAG5

**Fig 2:**
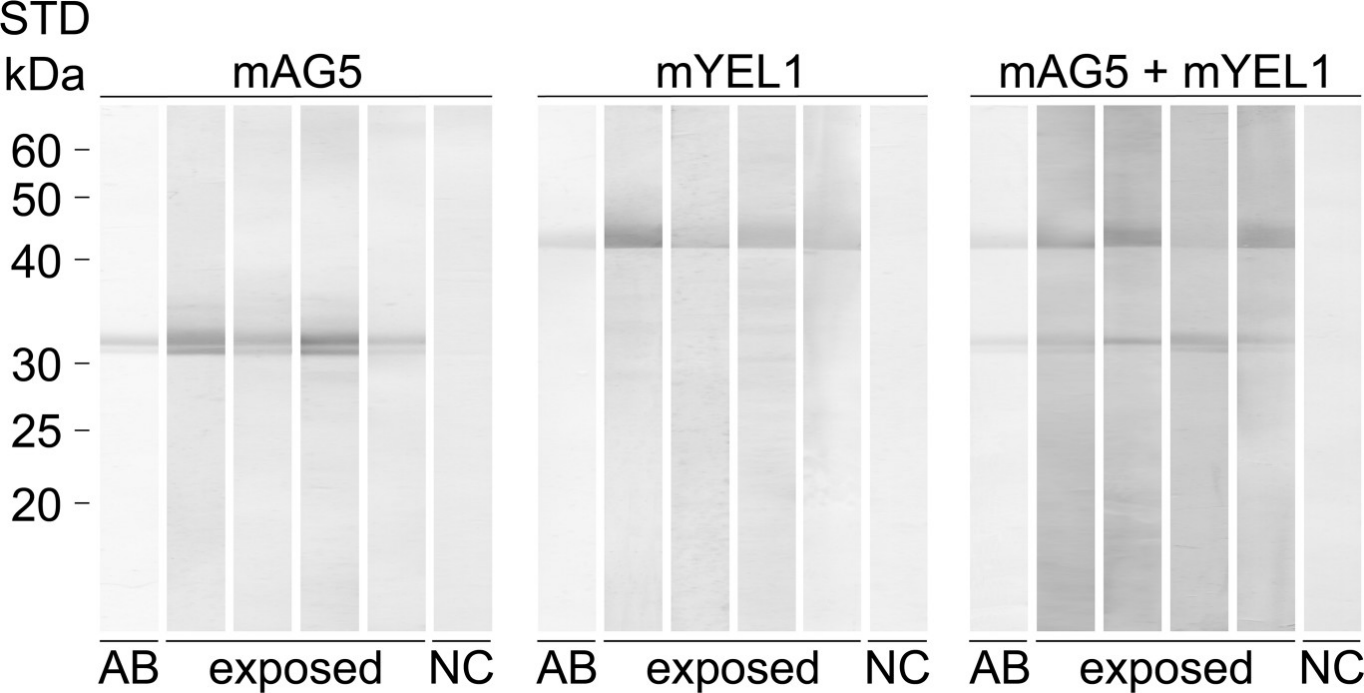
Antibody reaction against mYEL1, mAG5 and their combination on immunoblot. Recombinant mYEL1, mAG5, or both in equal amounts were run by SDS-PAGE under non-reducing conditions. Western blot analysis was performed for four human sera samples anti-recombinant proteins positive in ELISA (exposed) and one negative control serum (NC). AB stands for recombinant protein strip stained by amidoblack. Molecular weights (kDa) of standard (STD; BenchMark Protein Ladder, ThermoFisher Scientific) are indicated.

The relative intensity of antibody reactions against mYEL1, mAG5 and their combination is shown on an immunoblot (Fig 2). In total, four serum samples positive against both SGH and recombinant proteins in ELISA and one negative control serum were used. All anti-SGH positive samples reacted with both proteins and their combination. Western blot analysis showed that some serum samples recognize mYEL1 with more intensity than mAG5 or vice versa, reinforcing the use of combined antigens to enhance statistical variables of ELISA results.

### ELISA with mYEL1 and mAG5

IgG antibody response against SGH was compared by ELISA with response against mYEL1 and mAG5 (each one on its own or in combination). All Sudanese and Ethiopian samples were tested as well as negative control human sera.

For both recombinant proteins and their combination, the cut-off values were comparable with SGH (Table 2). The percentage of positive samples from both localities was highest for SGH, followed by mAG5, combination of mAG5 + mYEL1, and mYEL1 (Table 2). Statistical variables comparing the antibody reactions against all antigens (PPV, NPV, specificity, and sensitivity) achieved quite high values in case of PPV, NPV, and specificity (PPV ranged from 0.67 to 1, NPV ranged from 0.63 to 0.82, specificity ranged from 0.83 to 1), moderate values were achieved for sensitivity (range from 0.41 to 0.81) (Table 2). The highest values of all above mentioned statistical variables were achieved for the combination of mAG5 and mYEL1.

**Table 2:** Comparison of antibody reaction of human sera from Sudan, Ethiopia and both localities together against SGH, mAG5, mYEL1, and combination of both proteins.

	SGH			mAG5			mYEL1			mAG5 + mYEL1		
	S	E	S + E	S	E	S + E	S	E	S + E	S	E	S + E
**Cut-off**	0.238			0.145			0.335			0.284		
**Median**	0.25	0.19	0.19	0.10	0.11	0.11	**0.24**	**0.16**	**0.17**	**0.24**	0.15	0.16
**Q**_**1**_ **(25%)**	0.11	0.13	0.13	0.06	0.06	0.06	**0.11**	**0.10**	**0.10**	0.12	**0.10**	**0.10**
**Q**_**3**_ **(75%)**	0.58	0.29	0.32	0.22	0.17	0.17	0.36	**0.28**	**0.30**	**0.47**	0.27	**0.30**
**Positive (%)**	52.0	34.5	37.5	36.0	**31.1**	**31.9**	36.0	17.9	21.1	**44.0**	22.6	26.3
**PPV**	not applicable			**1.00**	0.67 **0.80**	0.74	0.78	0.79	0.78	0.96	**0.83**	**0.87**
**NPV**				0.75		0.79	0.63	0.75	0.73	**0.82**	0.78	**0.80**
**specificity**				**1.00**	0.84	0.87	0.83	**0.94**	**0.98**	0.96	**0.94**	0.94
**sensitivity**				0.69	**0.61**	**0.63**	0.54	0.41	0.44	**0.81**	0.54	0.61

Cut-off values, medians, Q_1_ (25^th^ percentile), Q_3_ (75^th^ percentile), positivity, positive and negative predictive values (PPV and NPV respectively), specificity, and sensitivity (true and false positive and negative values are available in S3 Table) for SGH, mAG5, mYEL1, and mAG5 + mYEL1 are indicated in this table. S, E and S + E represents Sudan, Ethiopia and both localities together, respectively. In total, 50 Sudanese, 235 Ethiopian, and 18 negative control human serum samples were used. The highest values or the best resemblance with SGH for each locality are written in bold.

**Fig 3:**
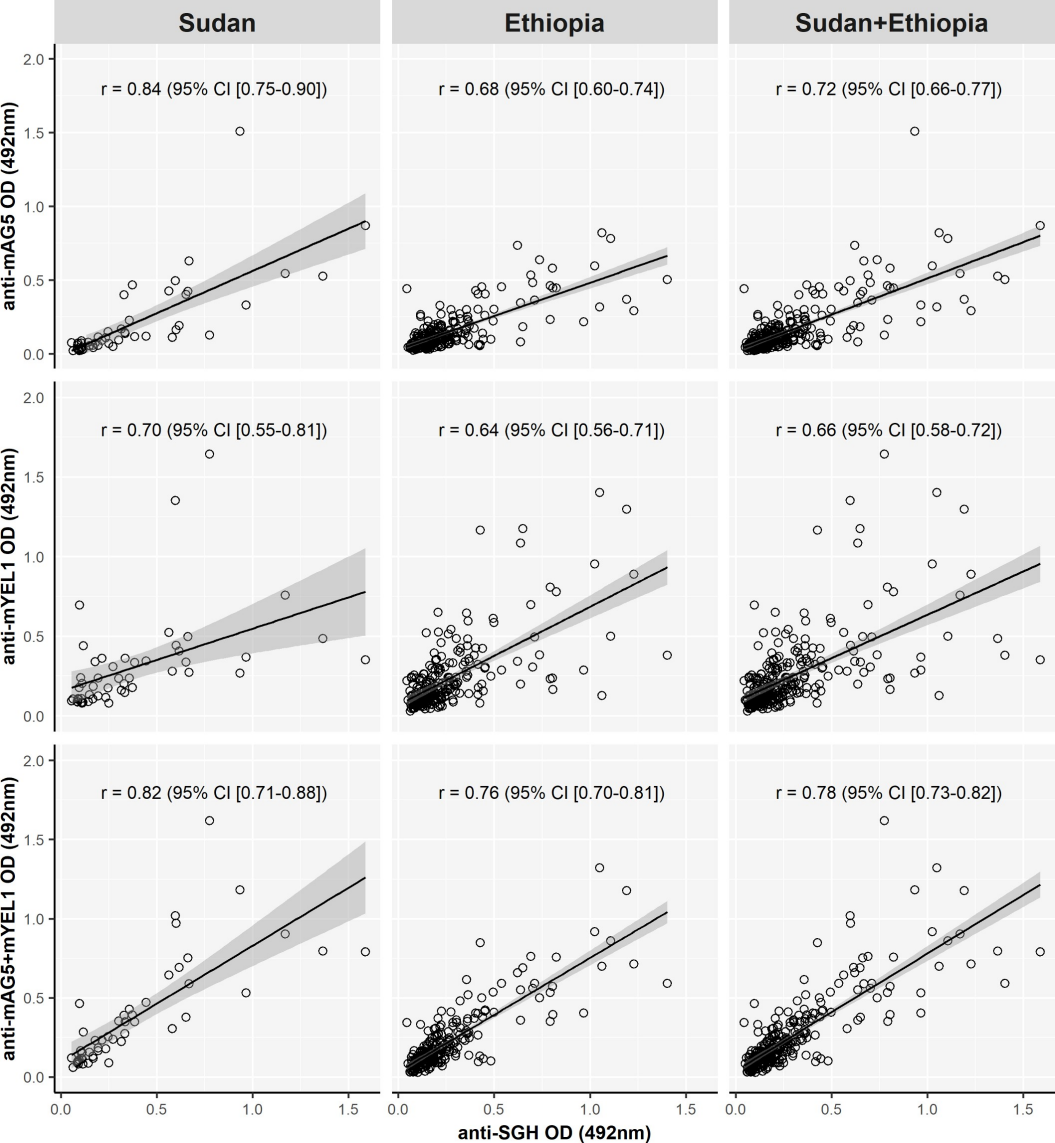
Correlations between IgG antibodies recognizing SGH and mAG5, mYEL1, or mAG5 + mYEL1 in human from Sudan and Ethiopia. Correlation between anti-SGH and anti-mAG5 (first row), between anti-SGH and anti-mYEL1 (second row) and between anti-SGH and combination of mAG5 and mYEL1 (third row) with Sudanese, Ethiopian, and serum samples from both localities together were performed by Spearman-Rank analysis. P value achieved in all analysis was below 0.0001. Correlation coefficients (r) and confidence intervals (CI) are indicated.

Correlation coefficients between anti-SGH antibody reaction and anti-mAG5 antibodies were slightly higher than coefficients between anti-SGH and anti-mYEL1 antibodies, with ranges from 0.68 to 0.84 and from 0.64 to 0.7, respectively. The highest correlation coefficients were achieved when both antigens were combined (range from 0.76 to 0.82) (Fig 3).

**Fig 4:**
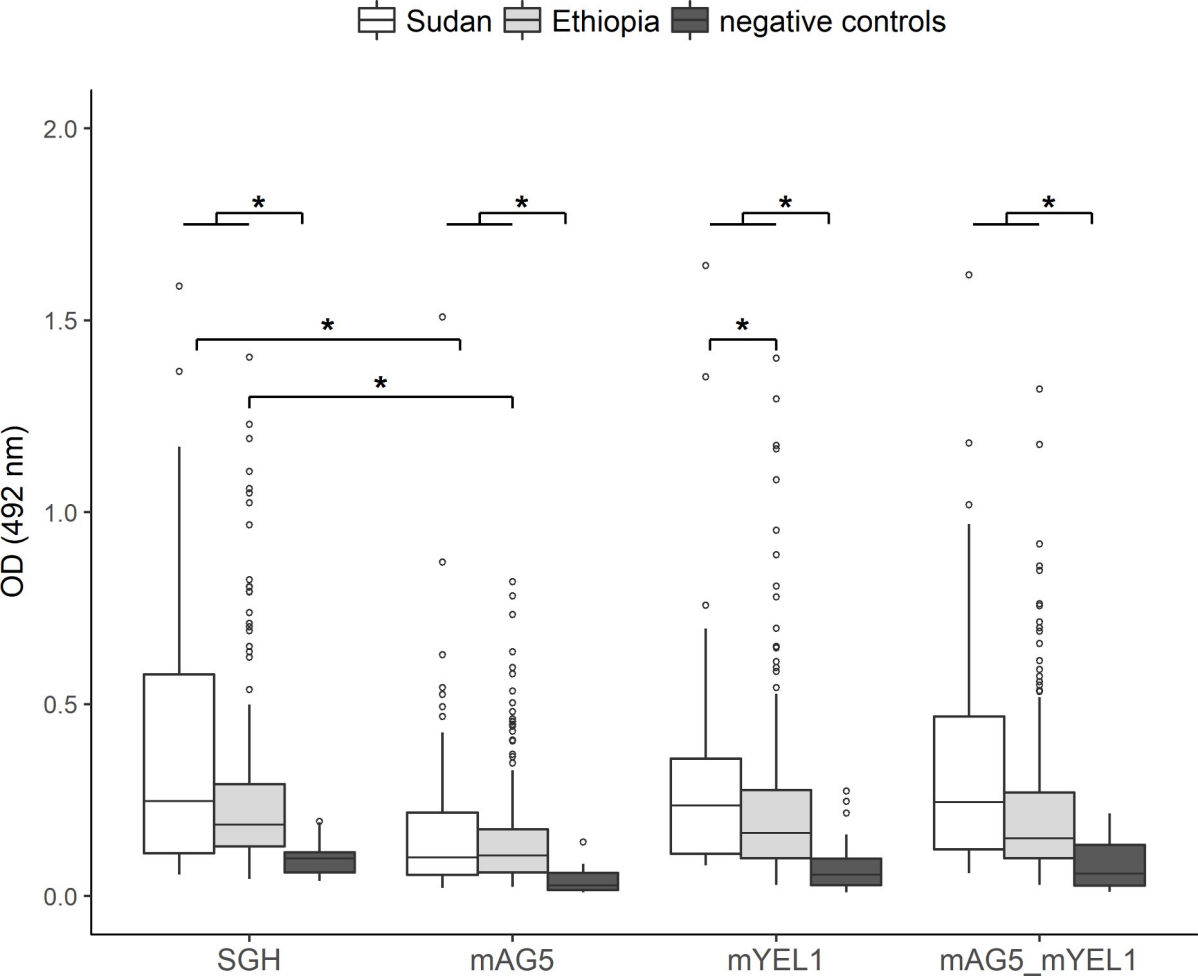
Antibody response against mAG5, mYEL1, their combination, and SGH. Plotted optical densities obtained from ELISA with serum samples from Sudan (50), Ethiopia (235), and 18 negative control samples. Results are shown in box plot graphs where boxes display the 25^th^-75^th^ percentiles of OD values and median value; vertical lines represent minimum to maximum values and dots the outliers. Asterisks notes statistical significance of p < 0.05.

Medians of antibody responses to mYEL1 and combination of mAG5 + mYEL1 correspond well to the ones achieved for SGH; while medians calculated for mAG5 were significantly lower (p < 0.004) (Fig 4). There was no statistical difference in levels of antibody response in Sudan versus in Ethiopia against SGH, mAG5 and mYEL1, even though medians calculated for SGH and mYEL1 were distinctly lower in Ethiopia than in Sudan (Fig 4). Significantly lower levels of IgG reaction in Ethiopia in comparison to Sudan were observed for combination of mAG5 and mYEL1 only (p = 0.02) (Fig 4).

## Discussion

Nine recombinant antigens (five expressed in *E*. *coli* and four expressed in human embryonic kidney cells) were tested as potential markers of anti-*P*. *orientalis* antibody response using human serum samples from Sudan and Ethiopia. Proteins, all belonging to the most abundant sand fly salivary protein families [30], were selected based on the immunoblot reactions with human sera and their known antigenicity for dogs or other domestic animals from Ethiopia, and for mice experimentally bitten by *P*. *orientalis* [11]. Yellow-related protein mYEL1, antigen 5-related protein mAG5 and especially their combination were shown to be promising candidates for replacing SGH in specific anti-*P*. *orientalis* salivary antigen IgG detection.

*Phlebotomus orientalis* is an only species from subgenus *Larroussious* which inhabits areas of interest. Other sand fly species present in both Sudan and Ethiopia include *P*. *papatasi* and *Sergentomyia schwetzi* which may bite humans but do not transmit visceral leishmaniasis [31]. Antibodies against salivary antigens of these two species do not cross-react with *P*. *orientalis* SGH or recombinant salivary proteins (bYEL1 and bAG5) confirming that they are species-specific [11]. We found 52% of the sera samples from Sudan and 34.5% samples from Ethiopia as anti-*P*. *orientalis* positive. The exposure and percentage of positive individuals was comparable or even higher than in other studies [21, 23]. The level of exposure to sand flies can be affected by many variables, including the closeness of the village to the vertisols (breeding sites of *P*. *orientalis*), the presence of domestic animals, the level of urbanization, the outdoor activities of the participants, etc. The lower positivity of participants from Ethiopia might be due to low numbers of *P*. *orientalis* in some of the villages where the samples were collected [8].

Proteins AG5, YEL1, and SP25 were expressed in both mammalian and bacterial systems. Although bPAR25 showed similar antigenic properties to mPAR25, both proteins were excluded from further analysis due to high cut-off values, low correlation coefficients with SGH, and big difference in samples positivity in comparison to SGH. In contrast with AG5 and YEL1 expressed in mammalian systems, the same antigens expressed in bacterial cells were not found antigenic for human. Different antigenic properties and low resemblance to anti-SGH antibody reaction in proteins expressed in bacterial system could be due to some *E*. *coli* impurities remaining in protein preparations even after purification process. These bacterial contaminants can lead to nonspecific serological reaction with anti-*E*. *coli* antibodies, which are present even in sera of healthy individuals [32]. As *E*. *coli* is highly prevalent in many African countries [33], anti-*E*. *coli* antibodies can be expected in a considerable part of the population. Since both antigen 5-related and yellow-related protein are in a native state glycosylated [30], other possible issue could be absence of glycosylation in bacterially expressed proteins, which could affect not only protein folding [34], but could also change their antigenic potential [35].

Antigen 5-related proteins were previously recognized as potent SGH antigens by sera of mice [30,36], dogs [37], rabbits [38], and hamsters [39] bitten by several Old World sand fly species. Recombinant antigen 5-related proteins were tested as potential markers of exposure in three sand fly species only: in *P*. *perniciosus*, antigen 5-related protein (expressed in *E*. *coli*) was not recognized by either sera from dogs or mice bitten by this sand fly [40] and in *P*. *orientalis*, bAG5 was specifically recognized by sera from experimentally bitten mice, but not by those from naturally exposed dogs, goats or sheep from Ethiopia [11]. In *L*. *intermedia*, recombinant antigen 5-related protein rLinB-13 expressed in mammalian system was specifically recognized by human sera from endemic areas of cutaneous leishmaniasis in Brazil [22]. Our study is therefore the first one describing recombinant antigen 5-related protein as a potent tool to screen exposure to Old World sand fly species in human. Recombinant mAG5 was specifically recognized in immunoblot and its ability to substitute SGH was verified in ELISA. The antibody reaction against mAG5 corresponds to response against SGH with a high correlation coefficient, similar to the one obtained for rLinB-13 in Brazil [22]. The mAG5 achieved high values of specificity, NPV, PPV, and moderate sensitivity. However, the median of antibody responses against mAG5 was significantly lower than the median of responses against SGH in both Sudan and Ethiopia. This finding suggests that the mAG5 cannot capture all antibodies aimed on SGH, thus reinforcing the use of mAG5 in combination with an additional antigen.

The antigenicity of yellow-related proteins was well recognized in various host and sand fly species in both Old and New World [11,15,37,38,41–46]. In *P*. *papatasi*, two yellow-related proteins, expressed in *E*. *coli*, were specifically recognized by sera of some experimentally bitten mice, but were not tested in any large scale survey [36]. In *L*. *longipalpis*, recombinant yellow-related proteins were produced in mammalian expression system. Proteins LJM11 and LJM17 were identified as suitable antigens to screen exposure in bitten people [21], while LJM11 only was shown also as a marker of exposure in chickens [46]. On contrary, human antibody response against recombinant yellow-related protein from related species *L*. *intermedia* did not correlate with response against SGH [22]. Recombinant yellow-related protein rSP03B (expressed in bacterial system) derived from saliva of *P*. *perniciosus*, a sand fly species closely related to *P*. *orientalis* was at first described as a potential candidate for measuring exposure in dogs and mice experimentally bitten by *P*. *perniciosus* [40]. Consequently, rSP03B was shown as a suitable tool to screen exposure in hares, rabbits and dogs naturally exposed in Spain, where anti-rSP03B antibody reaction strongly correlated with response against SGH [47]. A strong correlation between anti-rSP03B and anti-SGH antibody response was also achieved in dogs naturally exposed to *P*. *perniciosus* in Italy and Portugal [48,49]. Sero-strip based on rSP03B was able to rapidly screen dogs living in endemic areas for the presence of *P*. *perniciosus*, which made it the first feasible immunochromatographic test in the field of vector exposure [50]. It is worth mentioning, that protein rSP03B shares 86% homology to recombinant mYEL1 protein tested in our study. Recombinant bYEL1 from *P*. *orientalis* which was found non-antigenic to human subjects in our study, was shown as a potent tool to screen exposure to *P*. *orientalis* bites in dogs, goats, and sheep from Ethiopia [11]. The statistical variables describing anti-bYEL1 antibody response of sheep was similar to the ones obtained in our study for humans and mYEL1. The mYEL1 achieved high specificity and PPV; moreover median values measured for mYEL1 did not statistically differ to the ones measured for SGH. On the other hand, correlation coefficients, NPV, and sensitivity were in both Sudan and Ethiopia distinctly lower than for the other potent candidate, mAG5, thus further reinforcing combining both antigens.

Combining recombinant proteins mAG5 and mYEL1 in an ELISA test led to a distinct increase of correlation coefficients, PPV, and NPV. Correlation coefficient reached r = 0.8 in both Sudan and Ethiopia, a number comparable to studies carried out on dogs [11,47–49], but more promising than the coefficients previously achieved for human host [21,23]. Furthermore, the combination of mAG5 + mYEL1 showed non-significant difference from standard SGH ELISA. Similarly as it was for SGH and mYEL1, differences in medians of OD values between Sudan and Ethiopia were observed also for antigen combination. However this difference was significant only in case of combination mAG5 + mYEL1. This strengthens the conception of using this combination as a replacement for the SGH ELISA in further epidemiological studies. The utilization of combined antigens was further supported by western blot analysis showing that some serum samples recognize with more intensity mYEL1 and with less intensity mAG5 and vice versa. Two dominant antigens used together can therefore resemble in a better way the whole salivary glands, as was previously shown also for two recombinant yellow-related proteins from *L*. *longipalpis* [21]. On the contrary, in dogs naturally exposed to *P*. *perniciosus*, combining recombinant yellow-related protein with apyrase did not increase the performance of the yellow-related protein itself, even so it still attained quite high correlation with antibody response against SGH [48].

In conclusion, this study identified the combination of two major *P*. *orientalis* salivary antigens, yellow-related protein mYEL1 and antigen 5-related protein mAG5, as a valid tool to screen sand fly exposure in human from both Sudan and Ethiopia. This combination of stable recombinant proteins is able to substitute *P*. *orientalis* SGH and thereby to simplify studies aimed on detecting antibodies in bitten people in endemic areas of visceral leishmaniasis. On the basis of these results, protocols incorporating these recombinant proteins will be used to determine impact of large scale spraying of insecticides on lowering exposure to the bites of *P*. *orientalis* in Sudan. The trial will involve thousands volunteers from a hyperendemic region in Gedarif state, where above samples were obtained.

## Supporting information

S1 Fig Anti-*P*. *orientalis* IgG response against SGH in serum samples from Sudan and Ethiopia. Plotted optical densities obtained from ELISA with 50 Sudanese, 235 Ethiopian, and 18 negative control (NC) serum samples. Each circle represents one serum sample; dashed line represents cut-off value, black lines represents medians, asterisk denotes statistical significance (p < 0.0001).
(TIFF)

S2 Fig Antigens and sera dilution titrations. Summarized optical densities (ODs) of antibody reactions of three anti-*P*. *orientalis* SGH positive (empty circle) and two negative (full circle) human sera in two dilutions (indicated as S) with nine recombinant proteins in three concentrations (indicated as Ag; μg/well).
(TIF)

S1 Table Dilutions of antigens and sera for ELISA. Table of antigens and sera dilutions divided into four groups according to the order of experiments as mentioned above. For SGH, the concentration of 0.028 μg/well and the 1:100 dilution of sera was same in all sets of experiments. bRP and mRP stands for recombinant proteins expressed in bacterial and mammalian system, respectively.
(DOCX)

S2 Table Comparison of antibody reaction of SGH and recombinant proteins. Cut-off values, medians, positivity, and correlation coefficients between antibody response against bPAR25, mPAR25, mYEL1 in two serum dilutions (A stands for 1:50, B stands for 1:100), and antibody response against SGH are indicated in this table. In total, 25 serum samples were used for this experiment. NA stands for not applicable.
(DOCX)

S3 Table True and false positive and negative values. True positive, true negative, false positive and false negative values describing antibody reaction of all available human serum samples against SGH and recombinant proteins mAG5, mYEL1 and mAG5 + mYEL1 are indicated in this table. S, E and S+E represents Sudan, Ethiopia and both localities together, respectively. NA stands for not applicable.
(DOCX)
