# Supplementary figures and images for: Human antibody reaction against recombinant salivary proteins of Phlebotomus orientalis in Eastern Africa
Source: PLoS Negl Trop Dis. 2018 Dec 4;12(12):e0006981. doi: 10.1371/journal.pntd.0006981 (PMC6279015; doi:10.1371/journal.pntd.0006981)

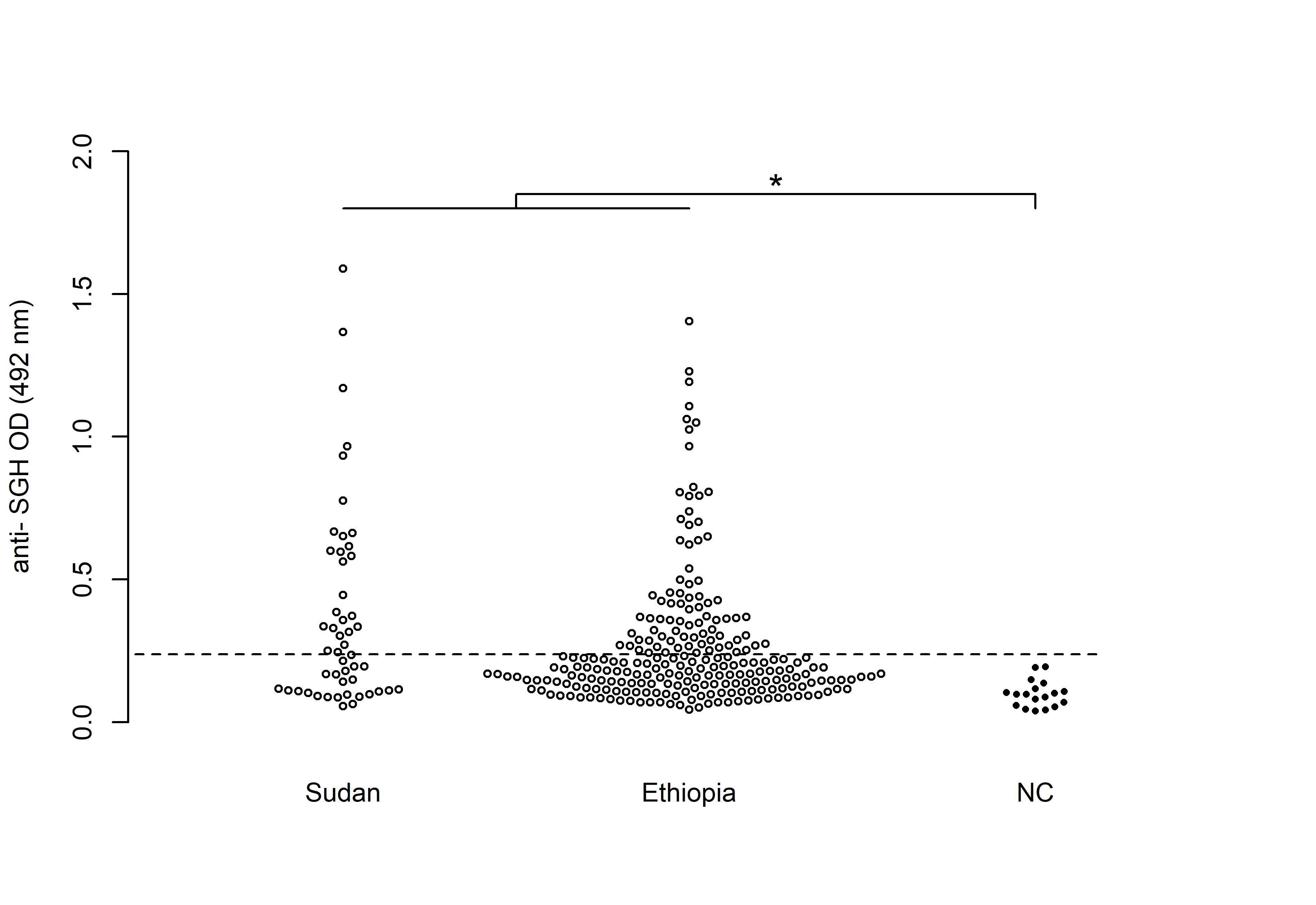

Supplement: S1 Fig — Plotted optical densities obtained from ELISA with 50 Sudanese, 235 Ethiopian, and 18 negative control (NC) serum samples. Each circle represents one serum sample; dashed line represents cut-off value, black lines represents medians, asterisk denotes statistical significance (p < 0.0001). (TIFF) [file pntd.0006981.s001.tiff]

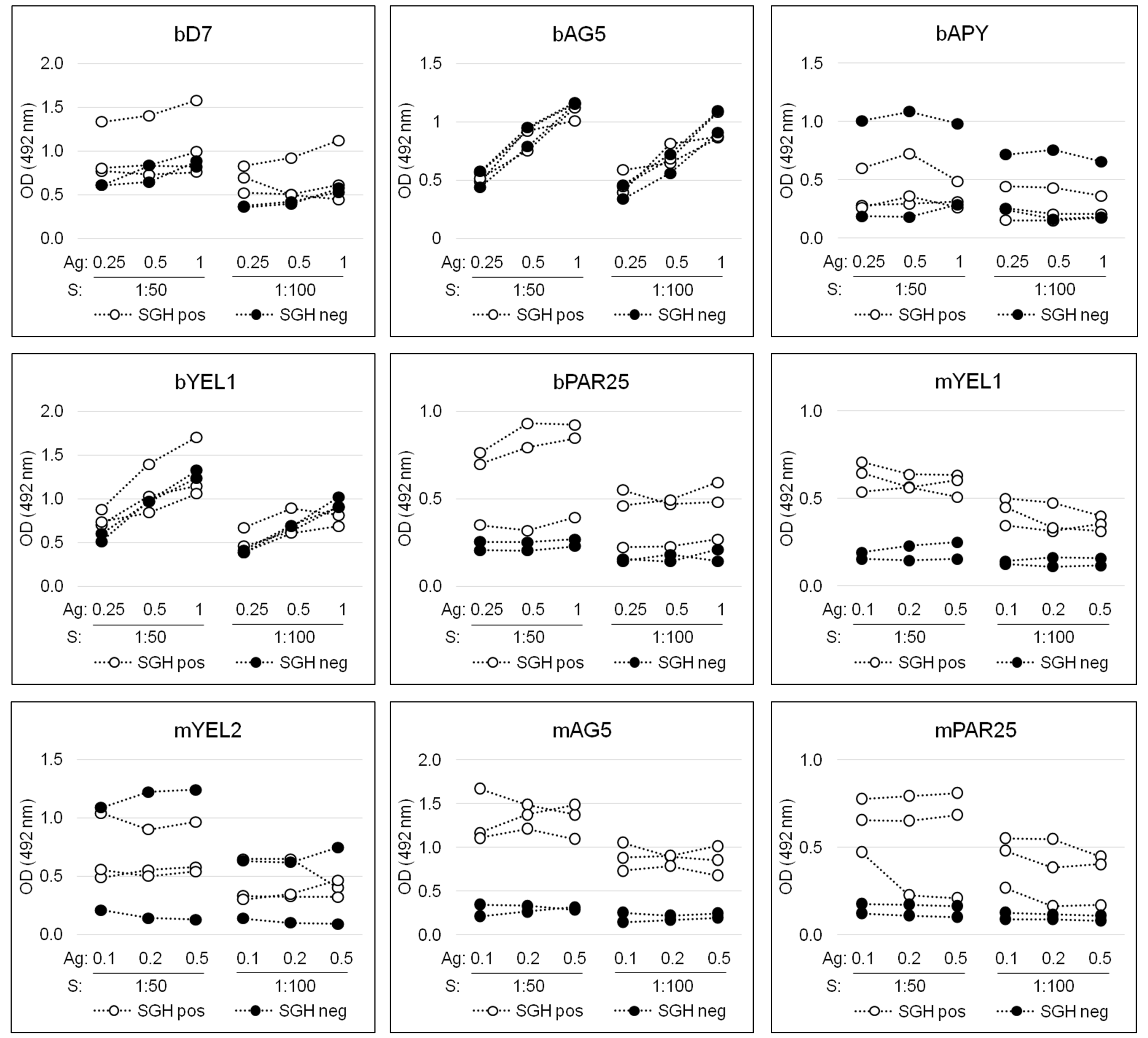

Supplement: S2 Fig — Summarized optical densities (ODs) of antibody reactions of three anti-P. orientalis SGH positive (empty circle) and two negative (full circle) human sera in two dilutions (indicated as S) with nine recombinant proteins in three concentrations (indicated as Ag; μg/well). (TIF) [file pntd.0006981.s002.tif]
