# Supplementary material for: Human antibody reaction against recombinant salivary proteins of Phlebotomus orientalis in Eastern Africa
Source: PLoS Negl Trop Dis. 2018 Dec 4;12(12):e0006981. doi: 10.1371/journal.pntd.0006981 (PMC6279015; doi:10.1371/journal.pntd.0006981)
Supplement: S1 Table — Table of antigens and sera dilutions divided into four groups according to the order of experiments as mentioned above. For SGH, the concentration of 0.028 μg/well and the 1:100 dilution of sera was same in all sets of experiments. bRP and mRP stands for recombinant proteins expressed in bacterial and mammalian system, respectively. (DOCX) [file pntd.0006981.s003.docx]

**S1 Table. Dilutions of antigens and sera for ELISA.**

|  | **1^st^ step** | **2^nd^ step** | | **3^rd^ step** | | | | **4^th^ step** | | | |
| --- | --- | --- | --- | --- | --- | --- | --- | --- | --- | --- | --- |
|  | **SGH** | **bRP** | **mRP** | **bPAR25** | **mPAR25** | **mYEL1** | **mAG5** | **mYEL1** | **mAG5** | | **mYEL1+mAG5** |
| **Antigen** (µg/well) | 0.028 | 0.25 | 0.1 | 1 | 0.2 | 0.1 | 0.1 | 0.1 | | 0.1 | 0.5 + 0.5 |
|  |  | 0.5 | 0.2 |  |  |  |  |  |  |  |  |
|  |  | 1 | 0.5 |  |  |  |  |  |  |  |  |
| **Serum** | 1:100 | 1:100 | 1:100 | 1:50 | 1:50 | 1:100 | 1:100 | 1:100 | | | |
|  |  | 1:50 | 1:50 |  |  | 1:50 |  |  |  |  |  |

Table of antigens and sera dilutions divided into four groups according to the order of ELISA experiments. For SGH, the concentration of 0.028 µg/well and the 1:100 dilution of sera was same in all sets of experiments. bRP and mRP stands for recombinant proteins expressed in bacterial and mammalian system, resp.
