# Supplementary material for: Human antibody reaction against recombinant salivary proteins of Phlebotomus orientalis in Eastern Africa
Source: PLoS Negl Trop Dis. 2018 Dec 4;12(12):e0006981. doi: 10.1371/journal.pntd.0006981 (PMC6279015; doi:10.1371/journal.pntd.0006981)
Supplement: S2 Table — Cut-off values, medians, positivity, and correlation coefficients between antibody response against bPAR25, mPAR25, mYEL1 in two serum dilutions (A stands for 1:50, B stands for 1:100), and antibody response against SGH are indicated in this table. In total, 25 serum samples were used for this experiment. NA stands for not applicable. (DOCX) [file pntd.0006981.s004.docx]

**S2 Table. Comparison of antibody reaction of SGH and recombinant proteins.**

|  | **SGH** | **bPAR25** | **mPAR25** | **mYEL1 (A)** | **mYEL1 (B)** | **mAG5** |
| --- | --- | --- | --- | --- | --- | --- |
| **Cut-off** | 0.155 | 0.796 | 0.451 | 0.280 | 0.175 | 0.240 |
| **Median** | 0.245 | 0.425 | 0.274 | 0.423 | 0.185 | 0.249 |
| **(min-max)** | (0.1-1.42) | (0.09-1.85) | (0.05-1.3) | (0.03-2.1) | (0.02-1.64) | (0.04-1.21) |
| **Positive (%)** | 80.0 | 35.0 | 25.0 | 65.0 | 70.0 | 65.0 |
| **Correlation** | NA | 0.47 | 0.70 | 0.80 | 0.78 | 0.91 |

Cut-off values, medians, positivity, and correlation coefficients between antibody response against bPAR25, mPAR25, mAG5, mYEL1 in two serum dilutions (A stands for 1:50, B stands for 1:100), and antibody response against SGH are indicated in this table. In total, 25 serum samples were used for this experiment. NA stands for not applicable.
