# Supplementary material for: Human antibody reaction against recombinant salivary proteins of Phlebotomus orientalis in Eastern Africa
Source: PLoS Negl Trop Dis. 2018 Dec 4;12(12):e0006981. doi: 10.1371/journal.pntd.0006981 (PMC6279015; doi:10.1371/journal.pntd.0006981)
Supplement: S3 Table — True positive, true negative, false positive and false negative values describing antibody reaction of all available human serum samples against SGH and recombinant proteins mAG5, mYEL1 and mAG5 + mYEL1 are indicated in this table. S, E and S+E represents Sudan, Ethiopia and both localities together, respectively. NA stands for not applicable. (DOCX) [file pntd.0006981.s005.docx]

**S3 Table. True and false positive and negative values.**

|  | **SGH** | | | **mAG5** | | | **mYEL1** | | | **mAG5 + mYEL1** | | |
| --- | --- | --- | --- | --- | --- | --- | --- | --- | --- | --- | --- | --- |
|  | **S** | **E** | **S+E** | **S** | **E** | **S+E** | **S** | **E** | **S+E** | **S** | **E** | **S+E** |
| **True positive** | 26 | 81 | 107 | 18 | 49 | 67 | 14 | 33 | 47 | 21 | 44 | 65 |
| **True negative** | 24 | 154 | 178 | 24 | 130 | 154 | 20 | 145 | 165 | 23 | 145 | 168 |
| **False positive** | NA | NA | NA | 0 | 24 | 24 | 4 | 9 | 13 | 1 | 9 | 10 |
| **False negative** | NA | NA | NA | 8 | 32 | 40 | 12 | 48 | 60 | 5 | 37 | 42 |

True positive, true negative, false positive and false negative values describing antibody reaction of all available human serum samples against SGH and recombinant proteins mAG5, mYEL1 and mAG5 + mYEL1 are indicated in this table. S, E and S+E represents Sudan, Ethiopia and both localities together, respectively. NA stands for not applicable.
